# Emphysema quantification using hybrid versus model-based generations of iterative reconstruction: SAFIRE versus ADMIRE

**DOI:** 10.1097/MD.0000000000014450

**Published:** 2019-02-15

**Authors:** Steve P. Martin, Joanna Gariani, Grégoire Feutry, Dan Adler, Wolfram Karenovics, Christoph D. Becker, Xavier Montet

**Affiliations:** a Diagnostic Department, Division of Radiology; b Department of Internal Medicine, Division of Pulmonology; c Department of Surgery, Division of Thoracic Surgery, Geneva University Hospital, Geneva, Switzerland.

**Keywords:** emphysema index, hybrid technique, iterative reconstruction, model-based technique, quantitative imaging

## Abstract

To compare 2 incompatible generations of iterative reconstructions from the same raw dataset based on automatic emphysema quantification and noise reduction: a hybrid algorithm called sinogram affirmed iterative reconstruction (SAFIRE) versus a model-based algorithm called advanced modeled iterative reconstruction (ADMIRE).

Raw datasets of 40 non-contrast thoracic computed tomography scanners obtained from a single acquisition on a SOMATOM Definition Flash unit (Siemens Healthcare, Forchheim) were reconstructed with 3 levels of SAFIRE and ADMIRE algorithms resulting in a total of 240 datasets. Emphysema index (EI) and image noise were compared using repeated analysis of variance (ANOVA) analysis with a *P* value <.05 considered statistically significant.

EI and image noise were stable between both generations of IR when reconstructed with the same level (*P* ≥0.31 and *P* ≥0.06, respectively).

SAFIRE and ADMIRE perform equally in terms of emphysema quantification and noise reduction.

## 1 Introduction

The widespread use of computed tomography (CT) has been contributing to the increase in radiation dose to the population since its inception in the 1970s. The number of CT scans has increased from 3 to 32 million between 1980 and 2007.^[1]^ Lowering the tube current-time product^[2,3]^ or tube potential^[4,5]^ were some of the strategies introduced to reduce the radiation dose delivered by CT scans. The technological advances and the increase in computational power allowed a renaissance of iterative reconstructions (IR), which were the initially proposed method for data reconstruction.^[6]^ IR has been shown to be a promising tool to lower radiation dose while maintaining diagnostic accuracy and quality imaging.^[7]^ sinogram affirmed iterative reconstruction (SAFIRE) and advanced modeled iterative reconstruction (ADMIRE) are the 2 latest IR algorithms released by Siemens Healthcare (Forchheim, Germany) in 2010 and 2015, respectively. iterative reconstruction in image space (IRIS) is their first-generation IR algorithm and will not be discussed in this paper.^[8]^ Studies on the impact of IR showed different results in the field of quantitative imaging such as emphysema assessment.^[9,10]^ IRs have been proven to influence emphysema quantification. Some of the studies evaluated the added value of IR technique in association with a dose-reduced protocol.^[11,12]^ Model-based reconstructions are offered as an alternative or a replacement of earlier generations based on a hybrid reconstruction technique.^[8]^ Hybrid techniques versus model-based techniques studies have already demonstrated that emphysema quantification is altered even more by the latest algorithm, at least for ASiR and MBIR (GE Healthcare, Waukesha, Wis.), respectively.^[13]^ As far as we know, no studies have been conducted in order to compare the 2 latest iterative algorithms from Siemens, namely SAFIRE and ADMIRE. This is probably because it is an almost impossible comparison. Once a system has been updated with ADMIRE, SAFIRE is no longer accessible. This comparison is only feasible on a prototype allowing to reconstruct raw data with both SAFIRE and ADMIRE. Hence, the primary goal of this study was to compare emphysema quantification using 2 IR algorithms. The secondary goals were to study image noise and segmentation on both IR.

## 2 Materials and methods

The local Ethics Committee on research involving humans approved this prospective study (CCER 15-048). Oral and written information was given and signed declarations of consent were obtained from all patients before examination.

### 2.1 Patients

Enrolment started on June 9 and finished on August 12, 2015. All consecutive patients undergoing a non-contrast thoracic CT scanner required clinically on the Somatom Definition Flash unit (Siemens Healthcare, Forchheim, Germany) of our department were included. Patients under 18 years of age and those who required intravenous contrast injection were excluded. The total study sample consisted of 58 patients. Four patients refused to participate. Fourteen CT examinations were excluded from the 3D quantitative analysis database due to image quality limiting automatic segmentation of the lungs. The final study sample consisted of 40 patients (M:F ratio 13:7, mean age 60 [range 18–89]).

### 2.2 Technical acquisition and reconstruction parameters and radiation dose

A single acquisition was performed craniocaudally during full inspiration from the apices to the bases of the lungs with the following parameters: collimation 64 × 2 × 0.6 mm, pitch 0.6, gantry rotation period 0.28 second, tube voltage 100 kV (CARE kV), tube current 120 mAs ref. (CARE Dose4D), slice thickness-interval 1 to 0.7 mm.

The raw data acquired on the VA44 system were reconstructed with 3 levels of IR of the 2 latest generations of algorithms, that is, SAFIRE 1, 3, 5 and ADMIRE 1, 3, 5.

Dose-length product (DLP) and CT dose index volume (CTDIvol) were obtained on the basis of a well-calibrated CT with a 32 cm phantom. Size-specific dose estimates (SSDE) were obtained via Bayer's Radimetrics^TM^ Enterprise Platform.

### 2.3 Image analysis

Automatic pulmonary segmentation of the lungs and emphysema quantification was performed using Pulmo3D (syngo.via VA30, Siemens), the reading and visualization software provided by the vendor. The volume of each lung was automatically calculated after lung segmentation. A threshold of -950 Hounsfield Units (HU) was applied to this volume to calculate the Emphysema Index (EI).

Electronic noise was assessed by collecting standard deviation values in HU with 3 standardized regions of interest (ROI) (∼1 cm^2^), 1 inside the trachea, 1 in the anterior extracorporeal air and 1 in the pectoral muscles. The mean value of the 3 measures was then considered as image noise. ROIs were carefully placed to avoid artefacts and clothes around the patients. Automatic propagation of the ROIs was performed in a copy-paste mode to assure the reproducibility of the location between the different reconstruction techniques.

### 2.4 Statistical analysis

The Gaussian distribution of the continuous variables of lung volume in liters, EI in percentage and image noise in HU was evaluated by the D’Agostino–Pearson omnibus normality test. When normality was confirmed, statistical differences were analyzed using a pairwise repeated measure (RM) 1-way analysis of variance (ANOVA) with the GreenHouse-Geisser correction and Tukey's multiple comparisons test. When normality was not confirmed, variables were analyzed using a pairwise Friedman test with Dunn's multiple comparisons test. A *P* value less than .05 was considered statistically significant. All variables were studied as means and standard errors of the mean.

## 3 Results

Six (SAFIRE 1-3-5, ADMIRE 1-3-5) different CT reconstructions from the same acquisition of 40 patients were objectively assessed, resulting in 240 datasets to be evaluated.

### 3.1 Radiation dose

The mean dose delivered was: DLP 225.3 ± 19.4 mGy.cm; CTDIvol was 6.41 ± 0.57 mGy; SSDE was 8.55 ± 0.61 mGy.

### 3.2 Quantitative analysis

**Figure 1:**
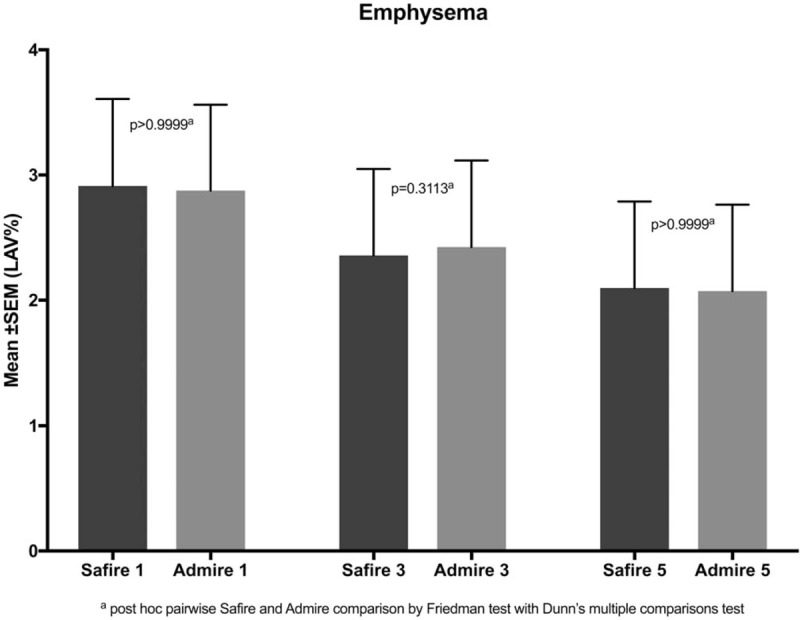
bars with error bars illustrating no significant differences in emphysema indexes between the same levels of the hybrid (SAFIRE) and the model-based (ADMIRE) generations of iterative reconstruction (*P* ≥.31). ADMIRE = advanced modeled iterative reconstruction, SAFIRE =  sinogram affirmed iterative reconstruction.

**Figure 2:**
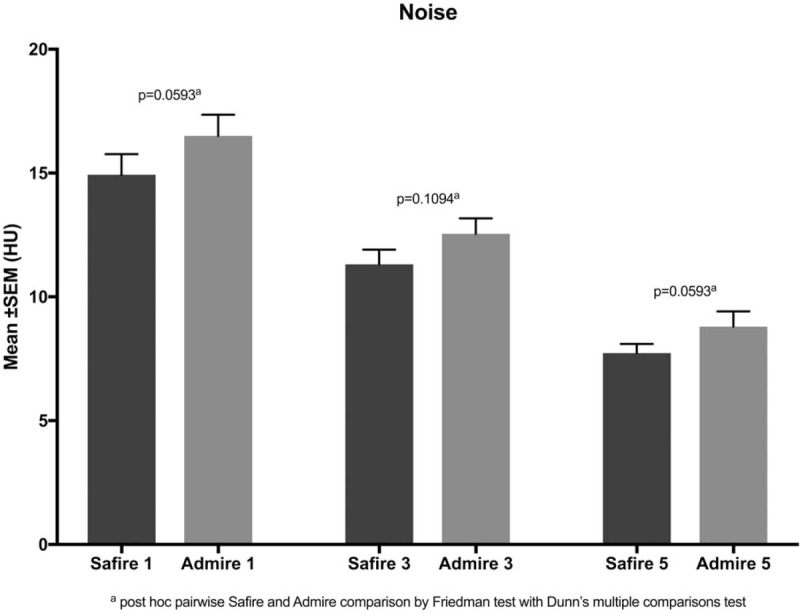
bars with error bars illustrating no significant difference in the reduction of image noise with the increase in levels of IR between the same levels of the hybrid (SAFIRE) and the model-based (ADMIRE) generations of iterative reconstruction (*P* ≥.06). ADMIRE = advanced modeled iterative reconstruction, SAFIRE =  sinogram affirmed iterative reconstruction.

**Figure 3:**
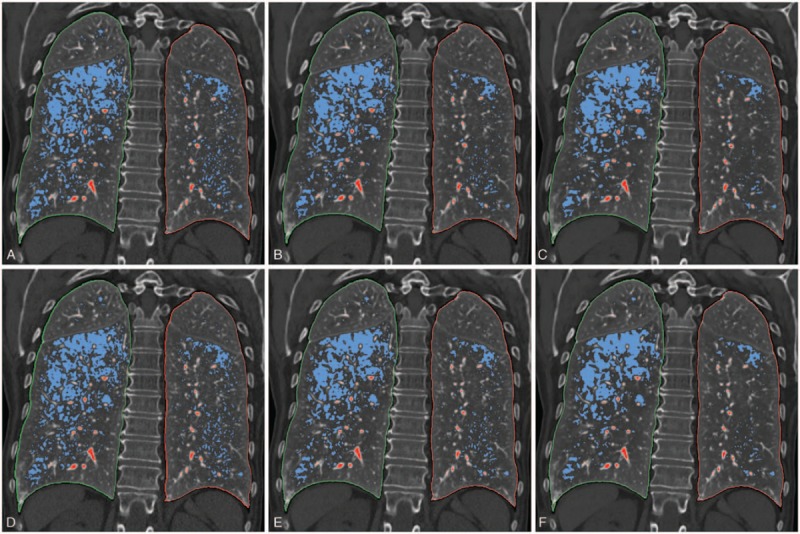
coronal reformatted images of unenhanced chest CT with blue-coloured overlays representing emphysematous lesions from SAFIRE 1 (A), SAFIRE 3 (B), SAFIRE 5 (C), ADMIRE 1 (D), ADMIRE 3 (E) and ADMIRE 5 (F). The lesser confluent blue areas reflect the similar reduction of emphysema index with the increase in the level of IR, best seen in the lower left lobe. ADMIRE = advanced modeled iterative reconstruction, SAFIRE =  sinogram affirmed iterative reconstruction.

Lung volume, EI and image noise are summarized in Table 1 and illustrated in Figures 1 to 3.

**Table 1:**
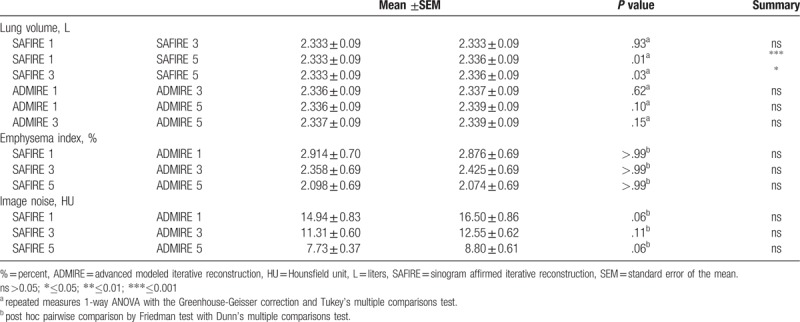
comparison of lung volume, emphysema index and image noise.

Lung volume comparison between the 3 levels of the same IR technique demonstrated no significant difference for ADMIRE (*P *≤* *.10). On the contrary, lung volume comparison between the 3 levels of SAFIRE showed no significant differences only for the levels 1 and 3 (*P* = .93).

The EI was not statistically different between SAFIRE and ADMIRE when reconstructed with the same level of IR (*P* >.99).

There was no significant difference in image noise when comparing the same levels of IR of SAFIRE and ADMIRE (*P *≥* *.06).

## 4 Discussion

Direct comparison of SAFIRE and ADMIRE is impossible on a clinical unit. Each IR algorithm has been developed on its own version of the system (VA44 for SAFIRE, VA48 for ADMIRE) and these are incompatible. Once the CT unit has been updated to VA48, ADMIRE is accessible, but unfortunately, SAFIRE no longer runs on that system. Raw data produced on a VA48 system cannot be loaded on a VA44 system and vice-versa. It is thereby impossible to compare SAFIRE and ADMIRE reconstructions without introducing a skew of acquisition or reconstruction. For this study, we used a VA44 compatible ADMIRE version.

The purpose of IR technique is to reduce image noise in order to allow a reduction in radiation while maintaining image quality. The dose reduction needs then to be matched to a certain level of IR to obtain an image quality similar to the gold standard obtain from standard radiation and classical reconstruction technique.^[7,10–12]^ The objective of our study was to evaluate independently the impact of the new model-based technique compared to the previous hybrid technique. Therefore, the design of our study did not require multiple acquisitions at different radiation doses.

Quantitative CT parameters including lung volume and EI play a relevant clinical role as predictors of mortality and morbidity. A study using mortality data collected during 8 years from the Norwegian Cause of Death Registry demonstrated that EI is a strong independent predictor of mortality with a shorter survival in patients with an EI ≥3% of 19 months.^[14]^ CT phenotypes can also help to classify patients with chronic obstructive pulmonary disease (COPD) at higher risk for exacerbations.^[15]^

Our study demonstrated that SAFIRE, the hybrid technique, and ADMIRE, the model-based technique, equally quantify emphysema when compared at the same strength of IR (Fig. 3). Keeping in mind the need for standardized CT protocols in the follow-up of patients with pulmonary emphysema or in longitudinal studies,^[16,17]^ it is relevant to point out that it is more important to be consistent with the levels of IR than with the type or the generation of algorithm when using either 1 of the 2 latest versions from Siemens.

ADMIRE had a denoising effect as effective as SAFIRE when the same level of IR was compared.

According to our study, ADMIRE had a lesser impact than SAFIRE on lung segmentation with lung volumes showing no statistical differences.

The major limitation of our study has already been identified as the use of a VA44 compatible version counterbalanced by the absence of skew of acquisition.

## 5 Conclusion

No statistical differences in emphysema quantification and image noise were shown between the 2 latest generations of IR algorithms. The added value of ADMIRE compared to SAFIRE lied in a statistically more robust segmentation of the lung. In other words, acquisition and reconstruction parameters do not need to be modified when upgrading from SAFIRE to ADMIRE in terms of emphysema quantification.

## Acknowledgments

The VA44 compatible version of ADMIRE was generously made available at Siemens Healthcare, Forchheim, Germany where Mr Thomas Allmendinger was a most welcoming host.

## Author contributions

**Conceptualization:** Steve P. Martin, Xavier Montet.

**Formal analysis:** Steve P Martin.

**Methodology:** Steve P Martin.

**Project administration:** Xavier Montet.

**Resources:** Christoph D Becker.

**Software:** Steve P Martin.

**Supervision:** Dan Adler, Wolfram Karenovics, Christoph D Becker, Xavier Montet.

**Validation:** Dan Adler, Wolfram Karenovics, Christoph D Becker, Xavier Montet.

**Writing – original draft:** Steve P Martin.

**Writing – review & editing:** Joanna Gariani, Grégoire Feutry, Christoph D Becker, Xavier Montet.

Steve P Martin orcid: 0000-0002-2318-2420.
